# Supplementary material for: Detection of 85 new active subglacial lakes in Antarctica from a decade of CryoSat-2 data
Source: Nat Commun. 2025 Sep 19;16:8311. doi: 10.1038/s41467-025-63773-9 (PMC12449472; doi:10.1038/s41467-025-63773-9)
Supplement: Supplementary file 1 — Supplementary Information [file 41467_2025_63773_MOESM1_ESM.pdf]

Supplement to **Detection of 85 new active subglacial lakes in Antarctica from a decade of CryoSat-2 data**

Sally F. Wilson et al., 2025.

Supplementary Figures 1-3

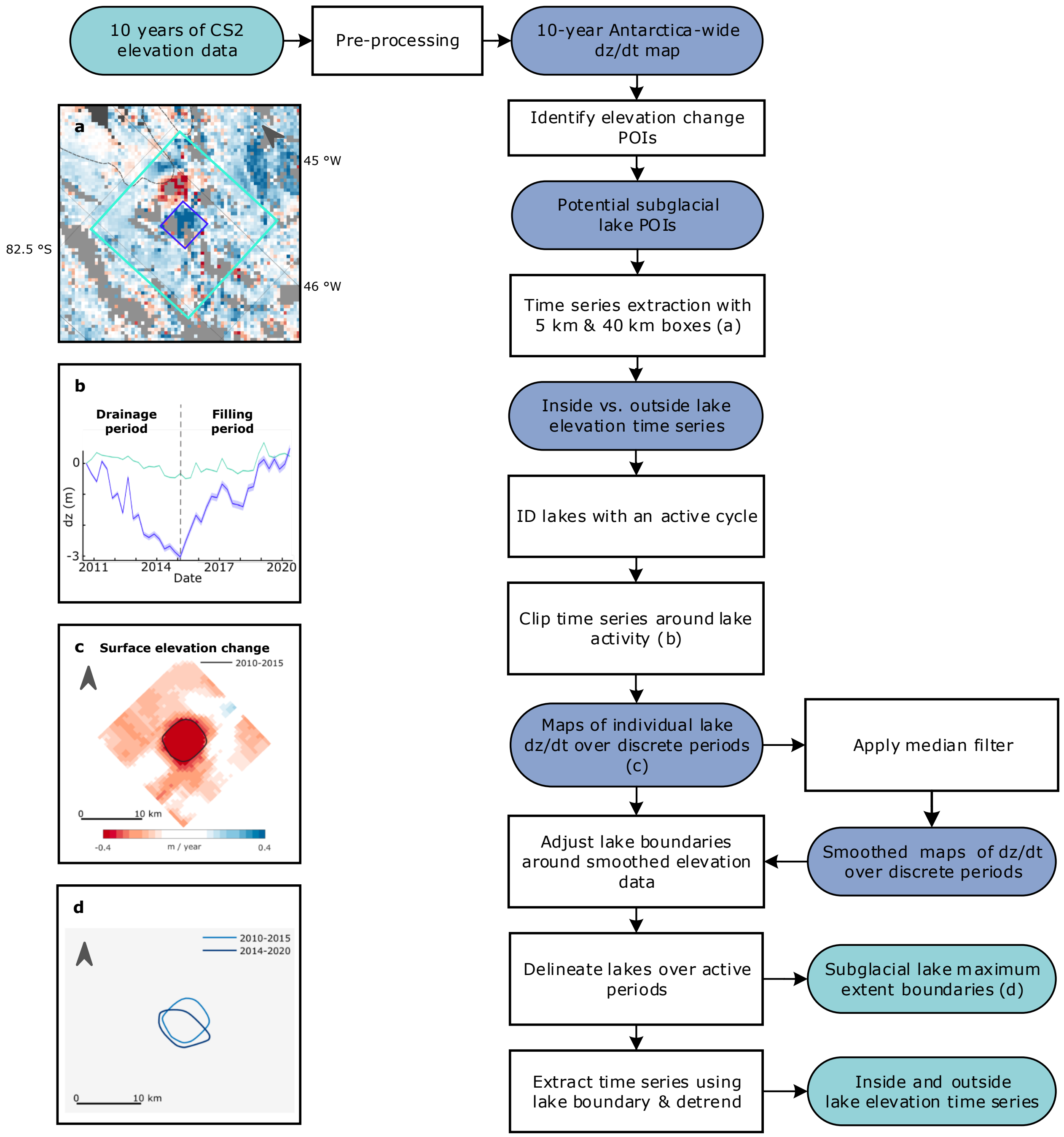

Supplementary Figure 1. **Flowchart detailing our method for detecting new subglacial lakes, delineating subglacial lake boundaries and extracting time series of elevation change.** Pre-processing covers computational steps discussed in section 2.2. Panels (a) through (d) illustrate processes within the diagram via maps over Support Force 3 subglacial lake, in East Antarctica.

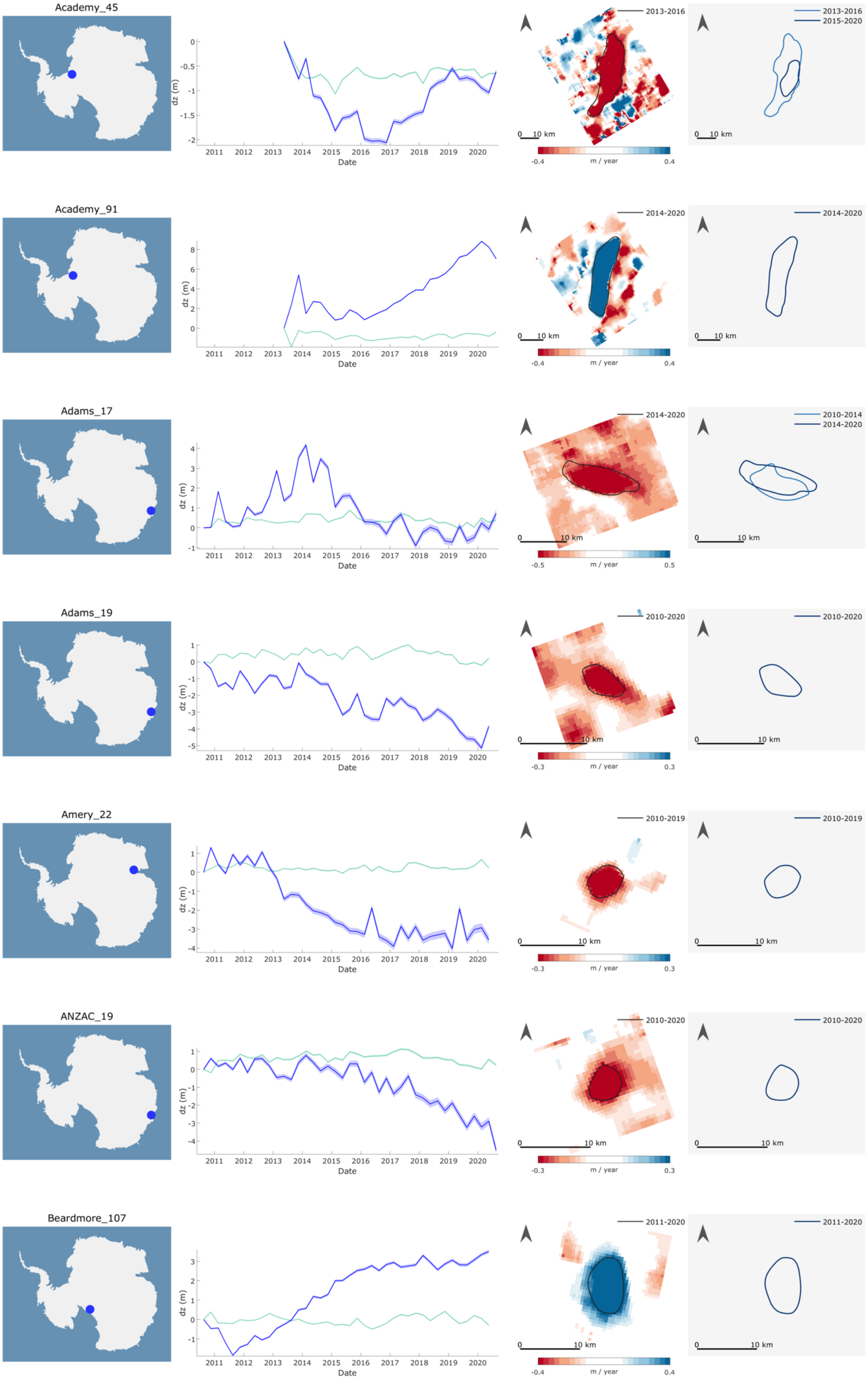

Academy_45
dz (m)
Date
2013-2016
2015-2020
0 10 km
-0.4 m / year 0.4
Academy_91
dz (m)
Date
2014-2020
0 10 km
-0.4 m / year 0.4
Adams_17
dz (m)
Date
2014-2020
2010-2014
0 10 km
-0.5 m / year 0.5
Adams_19
dz (m)
Date
2010-2020
0 10 km
-0.3 m / year 0.3
Amery_22
dz (m)
Date
2010-2019
0 10 km
-0.3 m / year 0.3
ANZAC_19
dz (m)
Date
2010-2020
0 10 km
-0.3 m / year 0.3
Beardmore_107
dz (m)
Date
2011-2020
0 10 km
-0.4 m / year 0.4
2011 2012 2013 2014 2015 2016 2017 2018 2019 2020

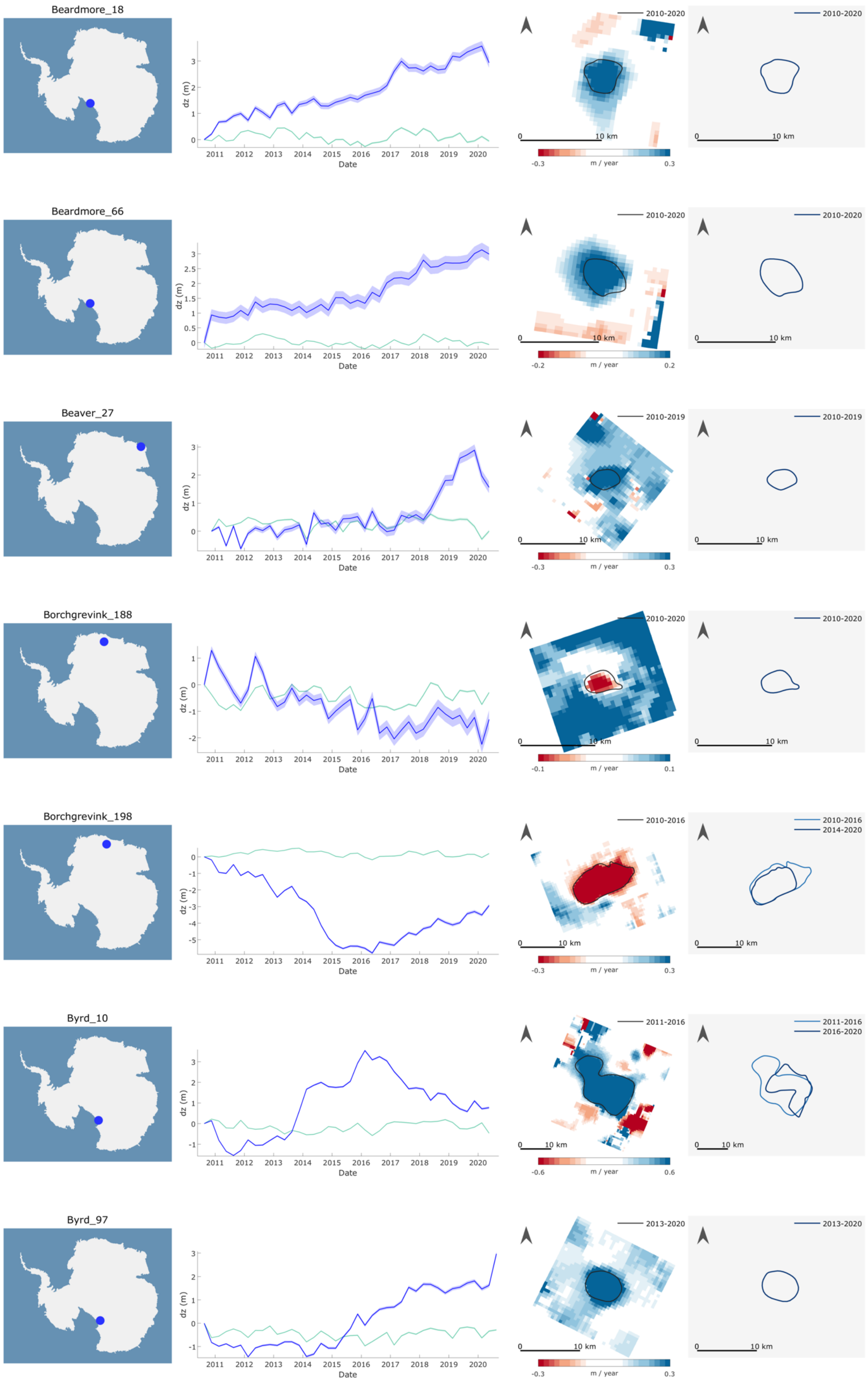

Beardmore_18
dz (m)
Date
2010-2020
10 km
m / year
Beardmore_66
2010-2020
Beaver_27
2010-2019
Borchgrevink_188
2010-2020
Borchgrevink_198
2010-2016
2014-2020
Byrd_10
2011-2016
2016-2020
Byrd_97
2013-2020

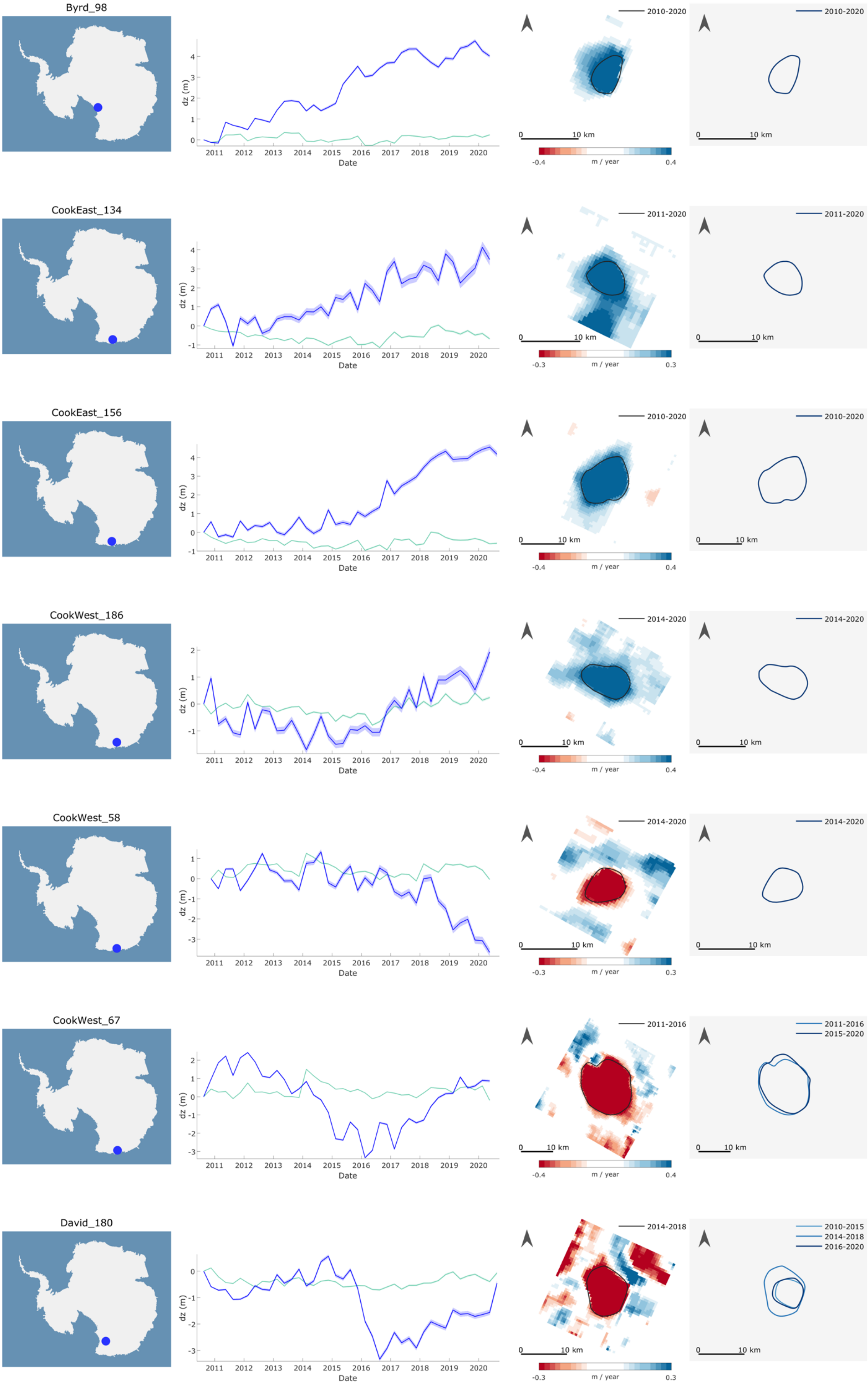
Byrd_98
CookEast_134
CookEast_156
CookWest_186
CookWest_58
CookWest_67
David_180
dz (m)
Date
2011 2012 2013 2014 2015 2016 2017 2018 2019 2020
2010-2020
2011-2020
2014-2020
2011-2016
2015-2020
2014-2018
2010-2015
2016-2020
0 10 km
m / year
-0.4 0.4
-0.3 0.3

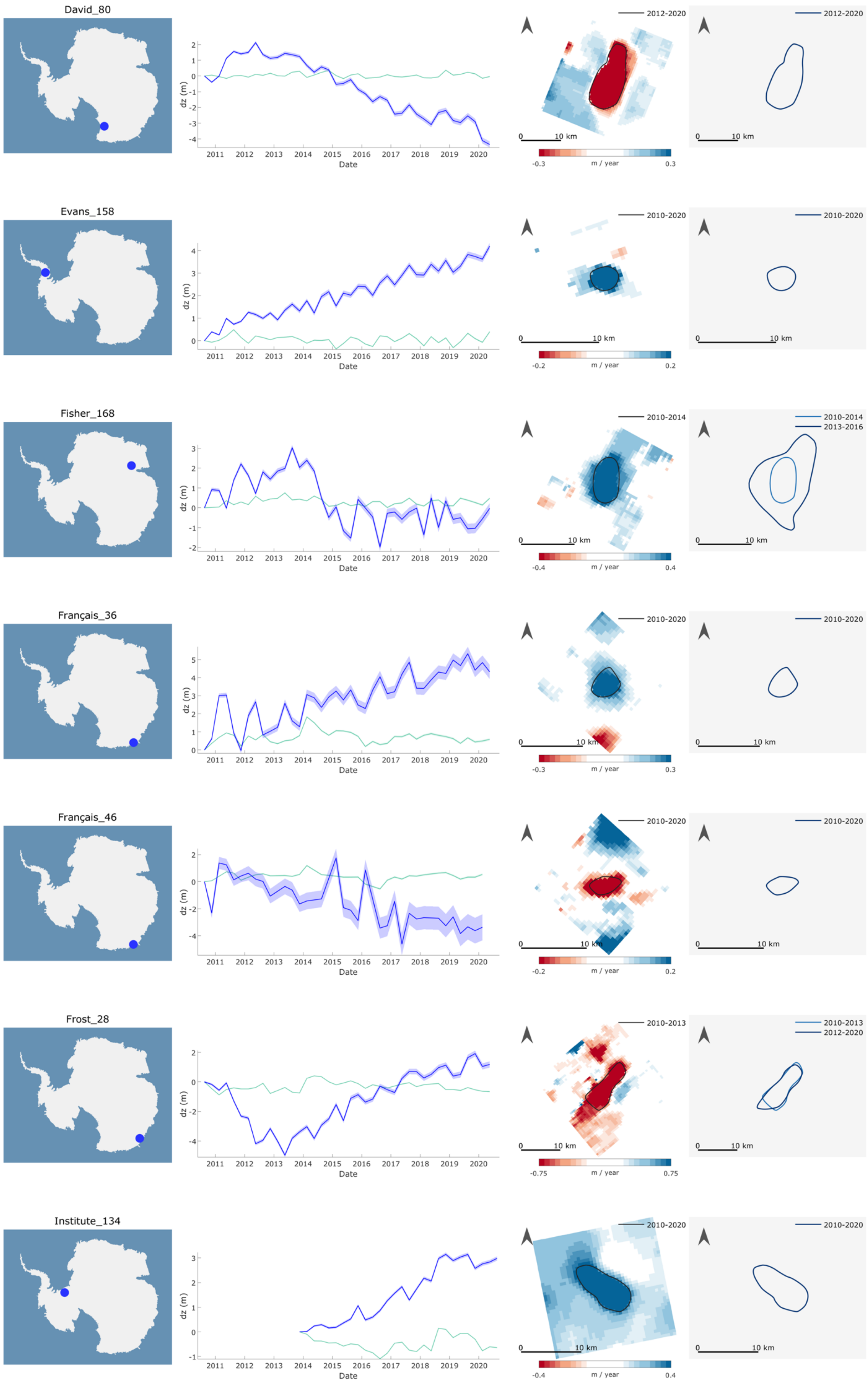
David_80
dz (m)
Date
2012-2020
10 km
-0.3
m / year
0.3
Evans_158
2010-2020
-0.2
0.2
Fisher_168
2010-2014
2013-2016
-0.4
0.4
Français_36
2010-2020
-0.3
0.3
Français_46
2010-2020
-0.2
0.2
Frost_28
2010-2013
2012-2020
-0.75
0.75
Institute_134
2010-2020
-0.4
0.4

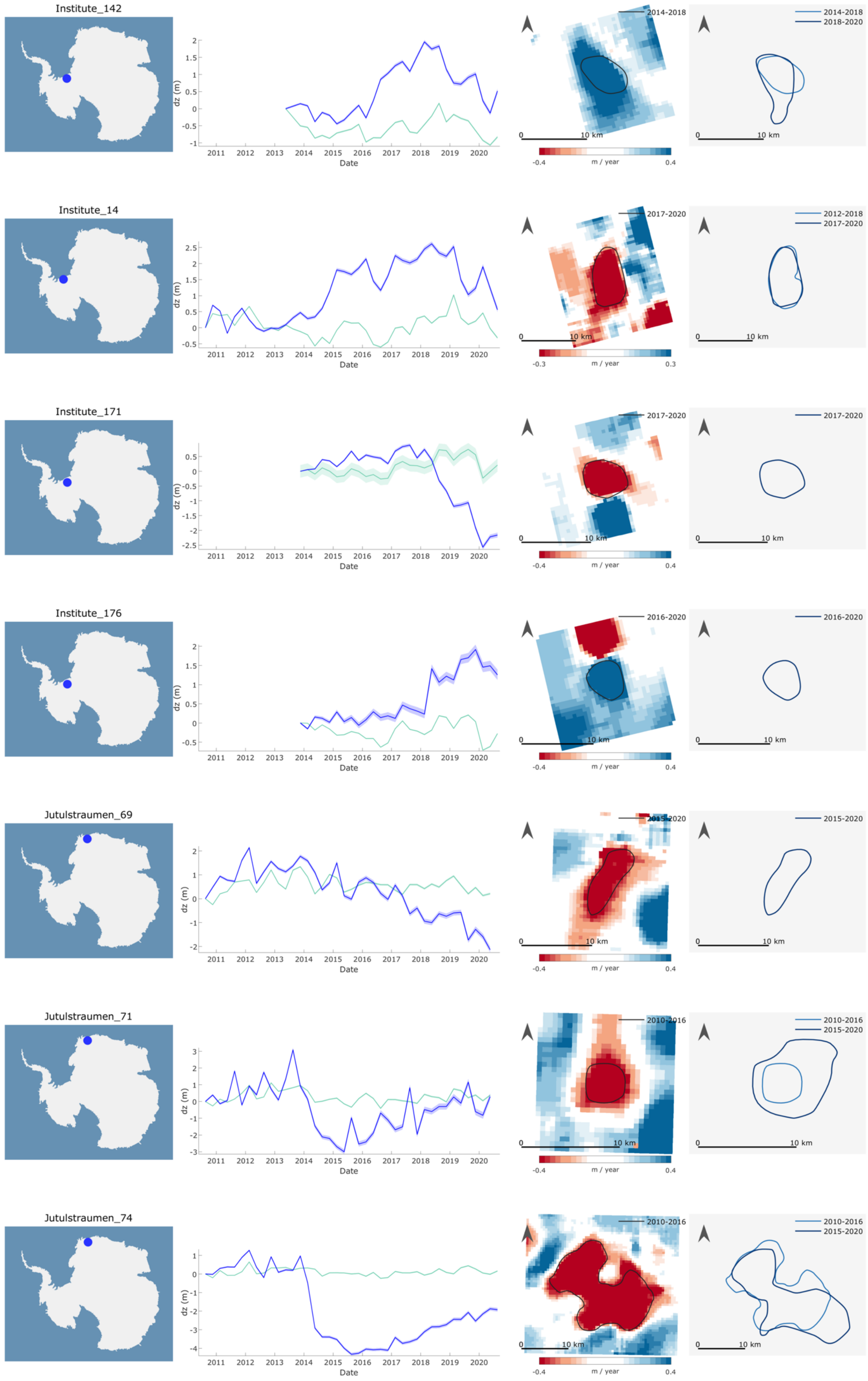
Institute_142
dz (m)
Date
2014-2018
2018-2020
10 km
m / year
Institute_14
2017-2020
2012-2018
Institute_171
Institute_176
2016-2020
Jutulstraumen_69
2015-2020
Jutulstraumen_71
2010-2016
Jutulstraumen_74

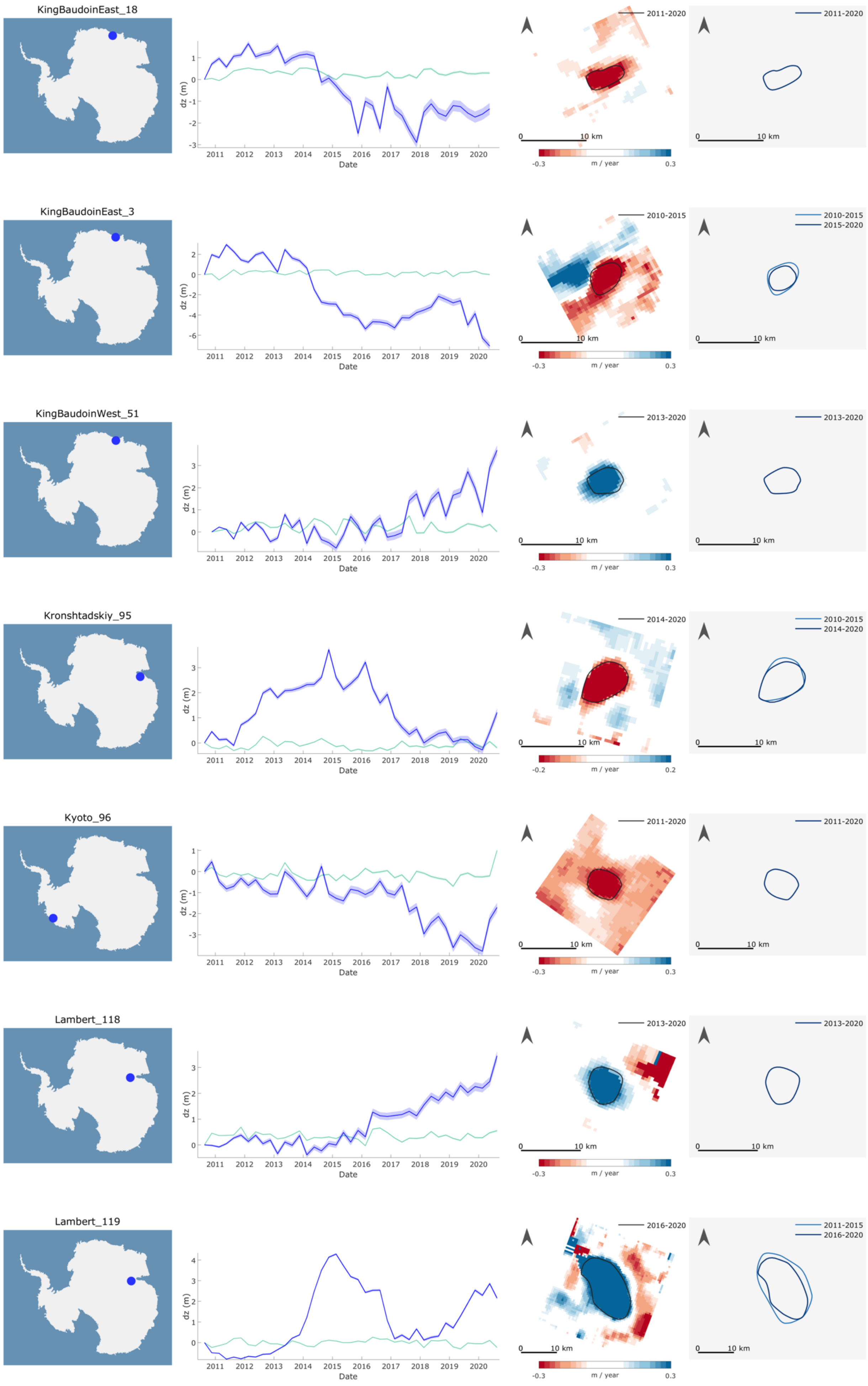
KingBaudoinEast_18
dz (m)
Date
2011-2020
0
10 km
-0.3
m / year
0.3
KingBaudoinEast_3
dz (m)
Date
2010-2015
2015-2020
0
10 km
-0.3
m / year
0.3
KingBaudoinWest_51
dz (m)
Date
2013-2020
0
10 km
-0.3
m / year
0.3
Kronshtadskiy_95
dz (m)
Date
2014-2020
2010-2015
0
10 km
-0.2
m / year
0.2
Kyoto_96
dz (m)
Date
2011-2020
0
10 km
-0.3
m / year
0.3
Lambert_118
dz (m)
Date
2013-2020
0
10 km
-0.3
m / year
0.3
Lambert_119
dz (m)
Date
2016-2020
2011-2015
0
10 km
-0.3
m / year
0.3
2011 2012 2013 2014 2015 2016 2017 2018 2019 2020

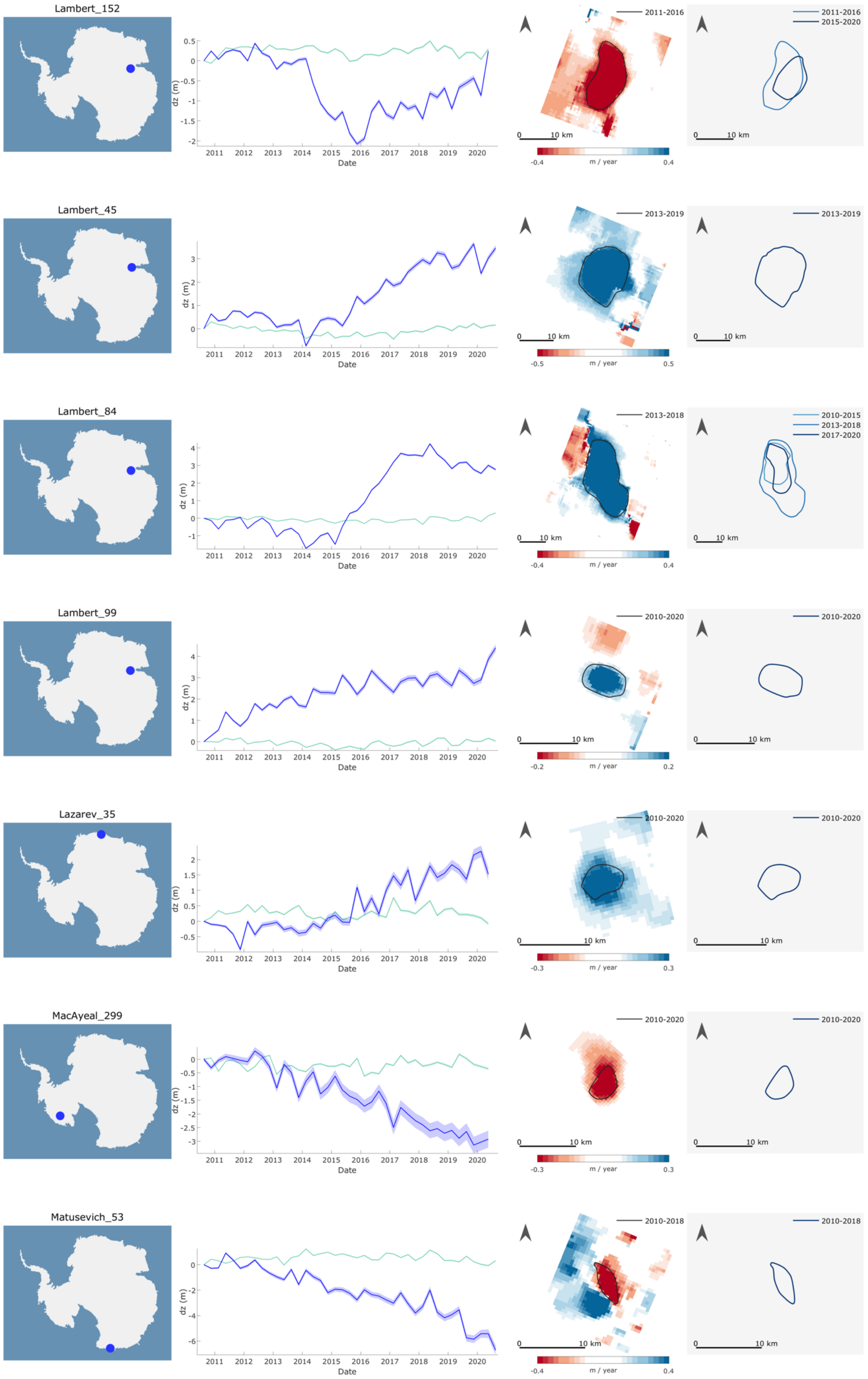

Lambert_152
dz (m)
Date
2011-2016
2015-2020
10 km
m / year
-0.4
0.4
Lambert_45
2013-2019
-0.5
0.5
Lambert_84
2013-2018
2010-2015
2017-2020
Lambert_99
2010-2020
-0.2
0.2
Lazarev_35
-0.3
0.3
MacAyeal_299
Matusevich_53
2010-2018
2011
2012
2013
2014
2015
2016
2017
2018
2019
2020

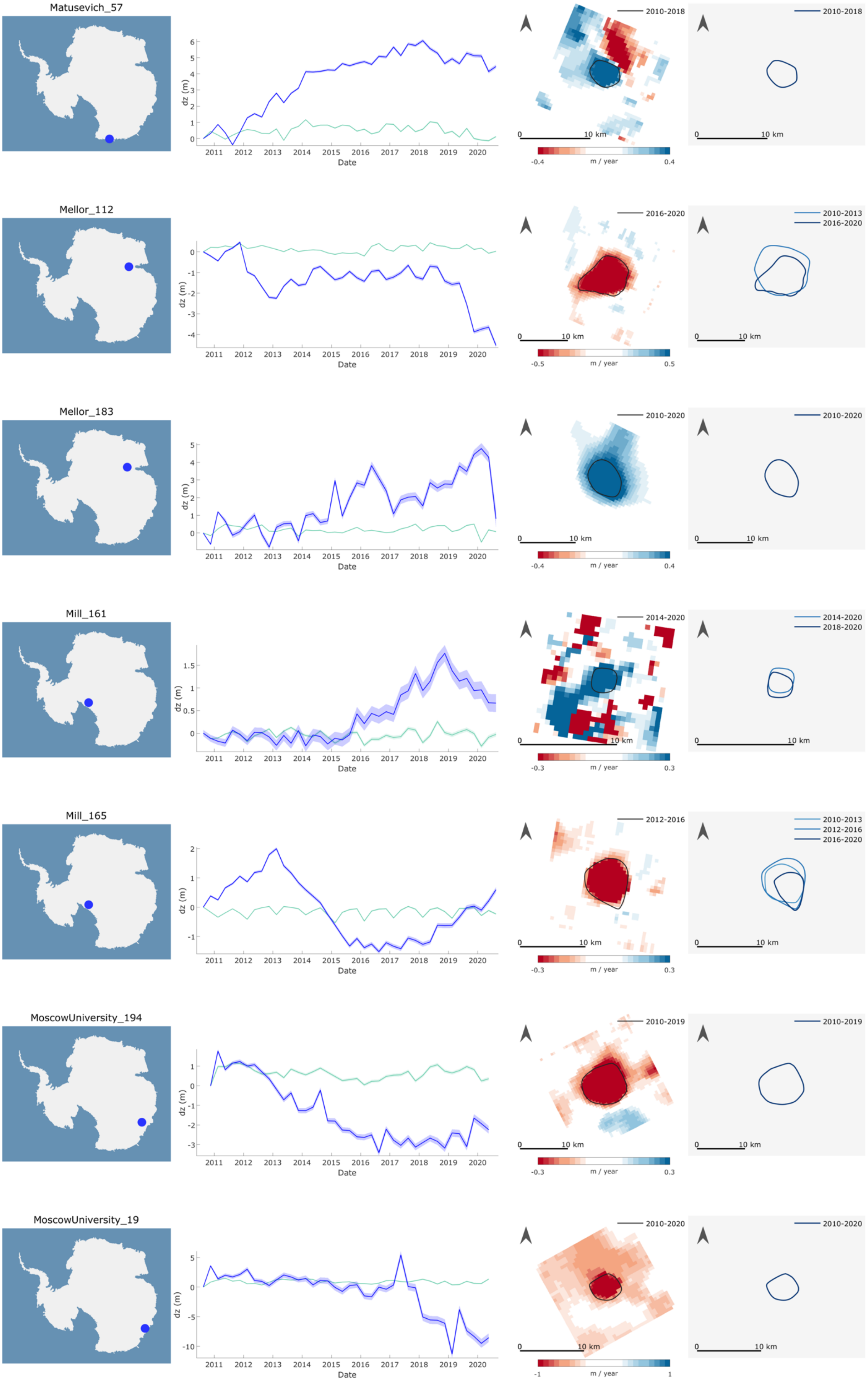

Matusevich_57
dz (m)
Date
2010-2018
0
10 km
-0.4
m / year
0.4
Mellor_112
2016-2020
2010-2013
-0.5
0.5
Mellor_183
2010-2020
Mill_161
2014-2020
2018-2020
-0.3
0.3
Mill_165
2012-2016
2016-2020
MoscowUniversity_194
2010-2019
MoscowUniversity_19
-1
1
2011
2012
2013
2014
2015
2016
2017
2018
2019
2020

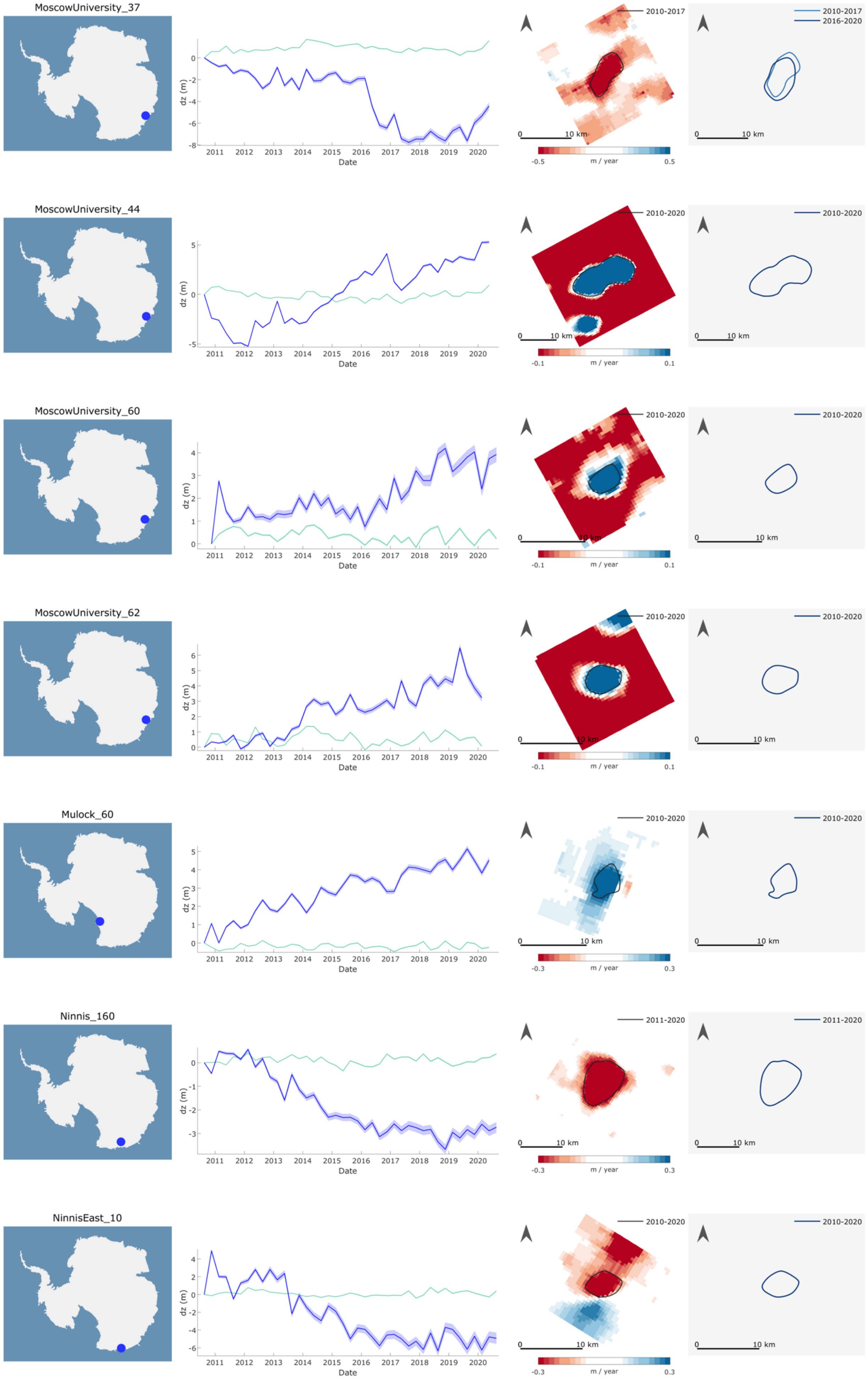

MoscowUniversity_37
dz (m)
Date
2010-2017
2016-2020
10 km
m / year
MoscowUniversity_44
2010-2020
MoscowUniversity_60
MoscowUniversity_62
Mulock_60
Ninnis_160
2011-2020
NinnisEast_10

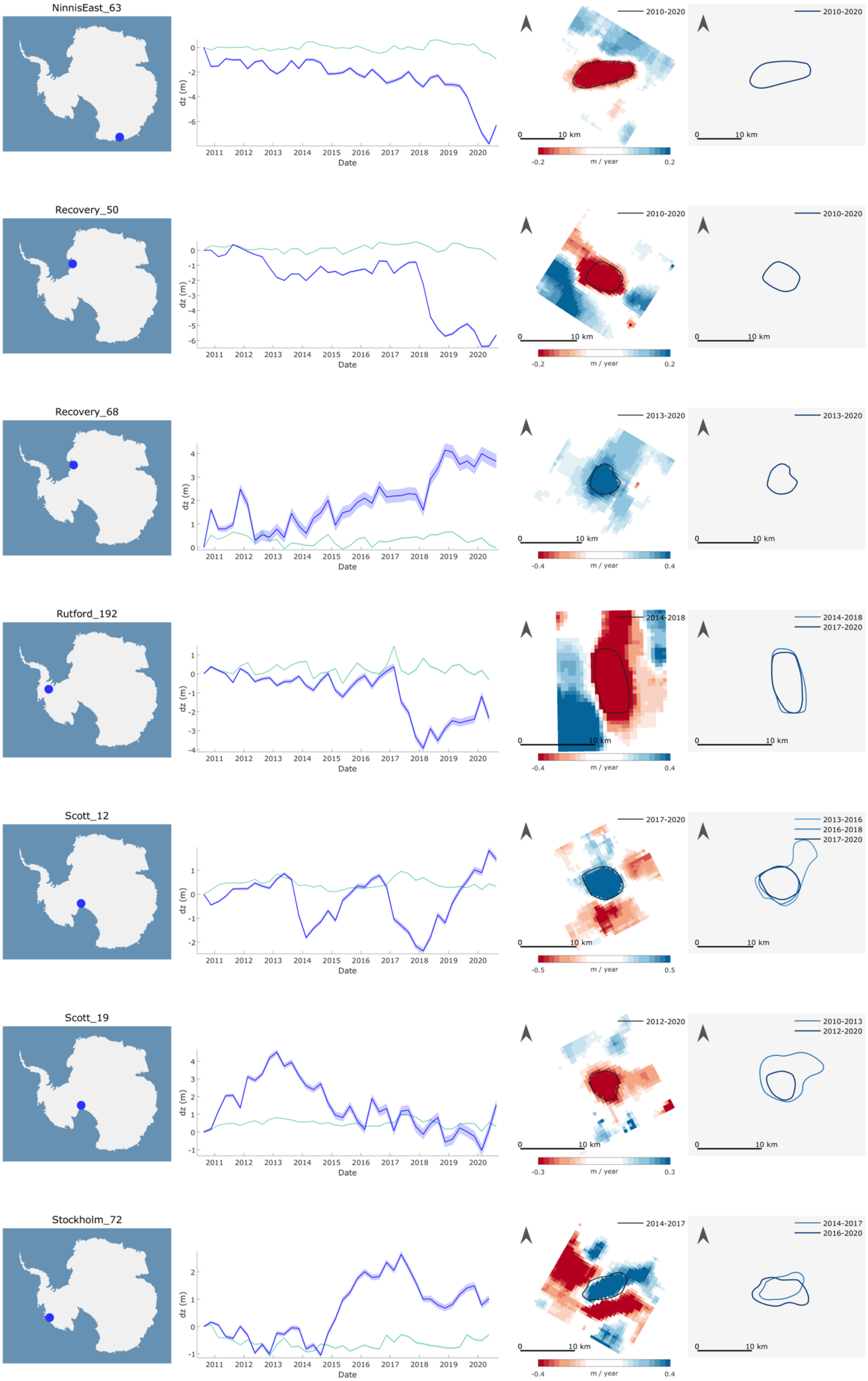

NinnisEast_63
dz (m)
Date
2011 2012 2013 2014 2015 2016 2017 2018 2019 2020
2010-2020
0 10 km
-0.2 m / year 0.2
Recovery_50
2010-2020
-0.2 m / year 0.2
Recovery_68
2013-2020
-0.4 m / year 0.4
Rutford_192
2014-2018
2017-2020
-0.4 m / year 0.4
Scott_12
2017-2020
2013-2016
2016-2018
-0.5 m / year 0.5
Scott_19
2012-2020
2010-2013
-0.3 m / year 0.3
Stockholm_72
2014-2017
2016-2020
-0.4 m / year 0.4

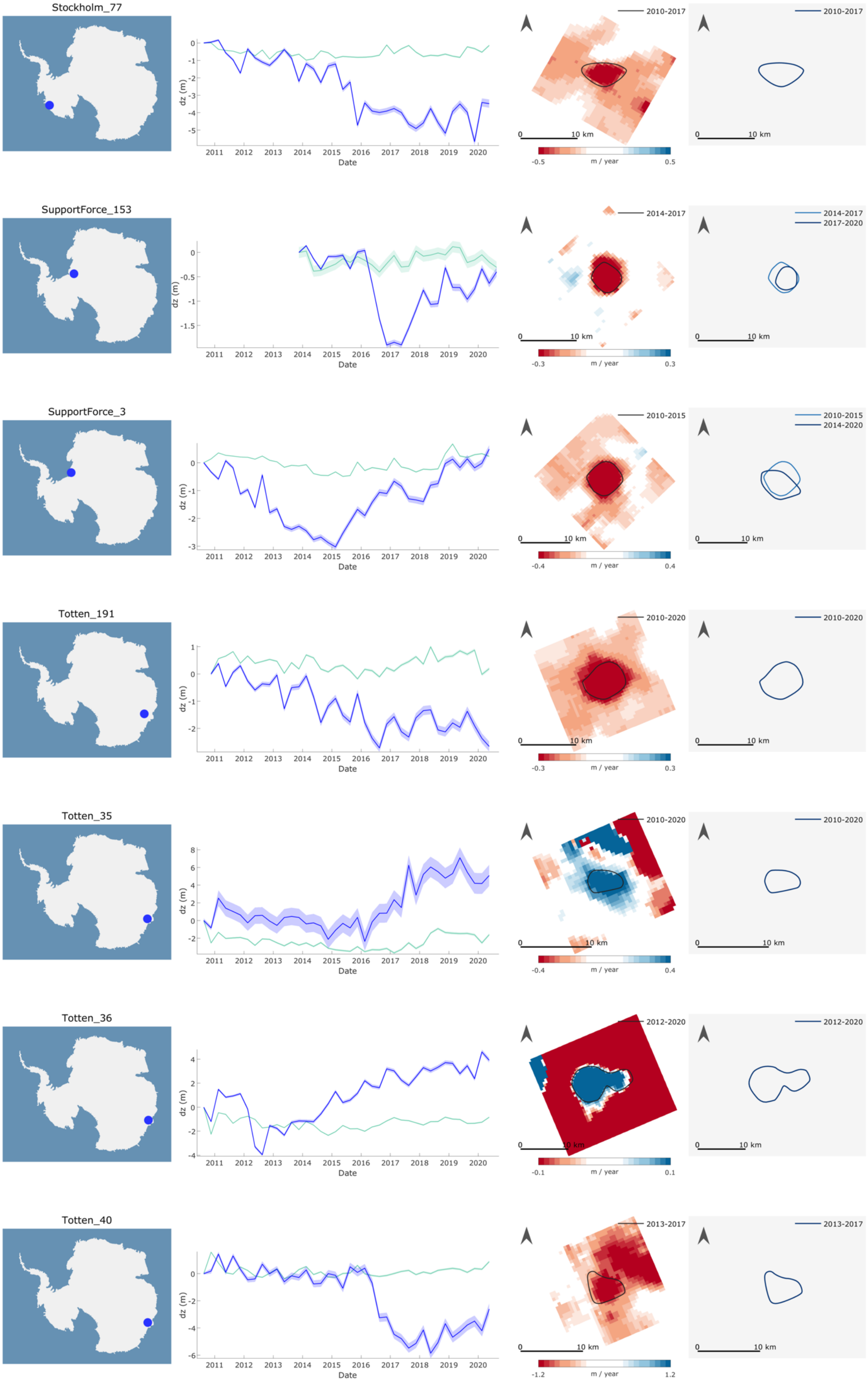
Stockholm_77
dz (m)
Date
2011
2012
2013
2014
2015
2016
2017
2018
2019
2020
2010-2017
10 km
m / year
-0.5
0.5
SupportForce_153
2014-2017
2017-2020
-0.3
0.3
SupportForce_3
2010-2015
2014-2020
-0.4
0.4
Totten_191
2010-2020
-0.3
0.3
Totten_35
2010-2020
-0.4
0.4
Totten_36
2012-2020
-0.1
0.1
Totten_40
2013-2017
-1.2
1.2

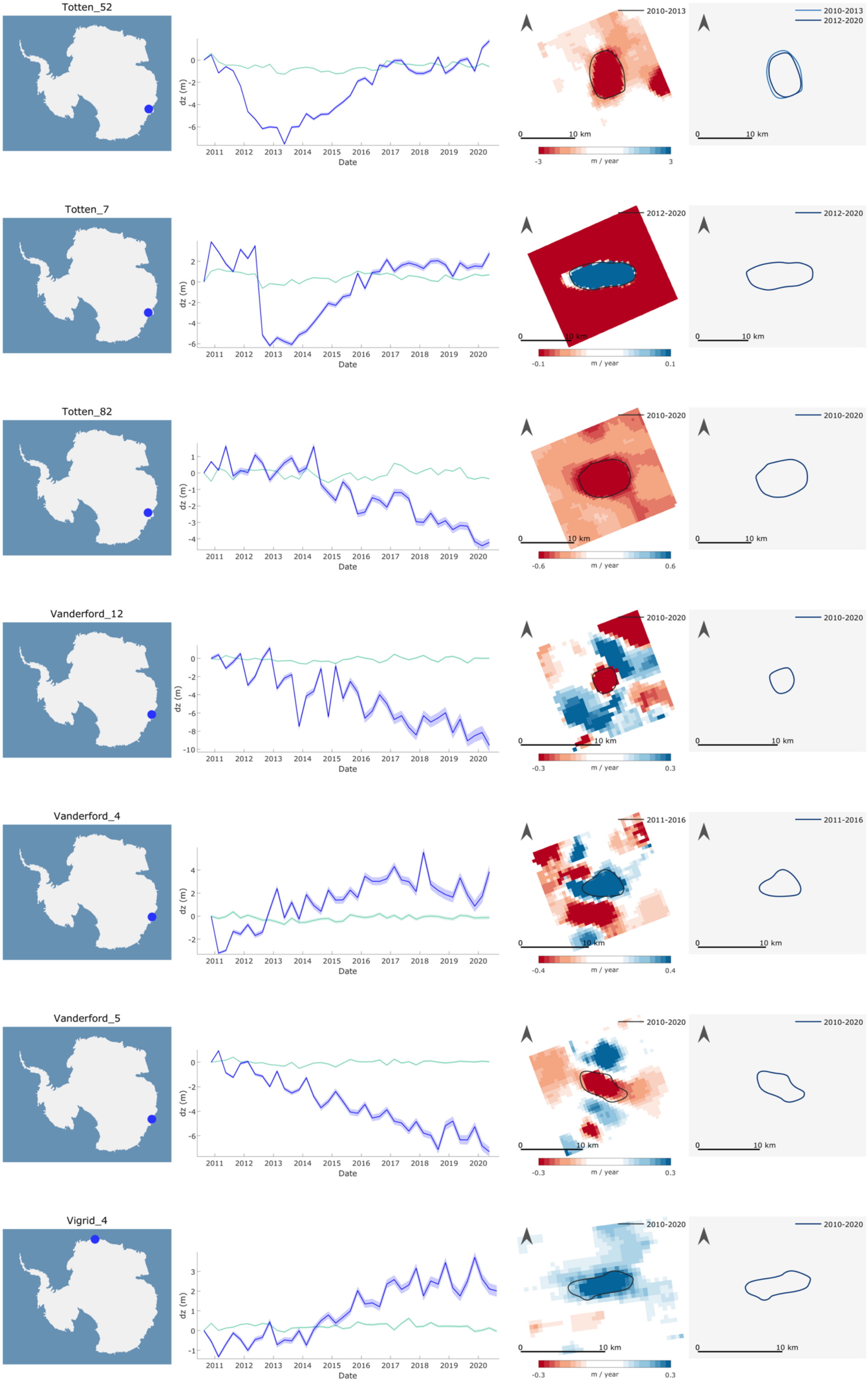

Totten_52
Totten_7
Totten_82
Vanderford_12
Vanderford_4
Vanderford_5
Vigrid_4
dz (m)
Date
2011
2012
2013
2014
2015
2016
2017
2018
2019
2020
2010-2013
2012-2020
2010-2020
2011-2016
0
10 km
m / year
-3
3
-0.1
0.1
-0.6
0.6
-0.3
0.3
-0.4
0.4

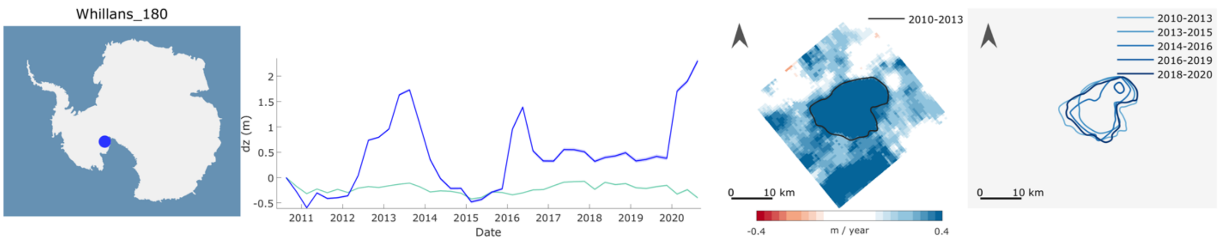

Supplementary Figure 2. **Location map, elevation change time series, an elevation change map and time-variable subglacial lake boundaries for all 85 new subglacial lakes (from left to right).** For each subglacial lake (row) we show: (a) a map showing the subglacial lake location (blue dot) on the Antarctic Ice Sheet. (b) Three-month mean timeseries of ice surface elevation change (dz) from 2010 to 2020, with subglacial lake elevation change (blue line) compared to elevation change from non-subglacial lake region (green line). Cumulative error is shown by the shaded area around each time series. (c) A surface elevation change map is shown for one event on each subglacial lake, with the corresponding lake boundary delineated from these data (black line). Time periods for these maps and boundaries are illustrated in the top right corner, and the North direction is indicated by the grey arrow. (d) Time-variable subglacial lake boundaries (light to dark blue lines) are delineated for each active phase.

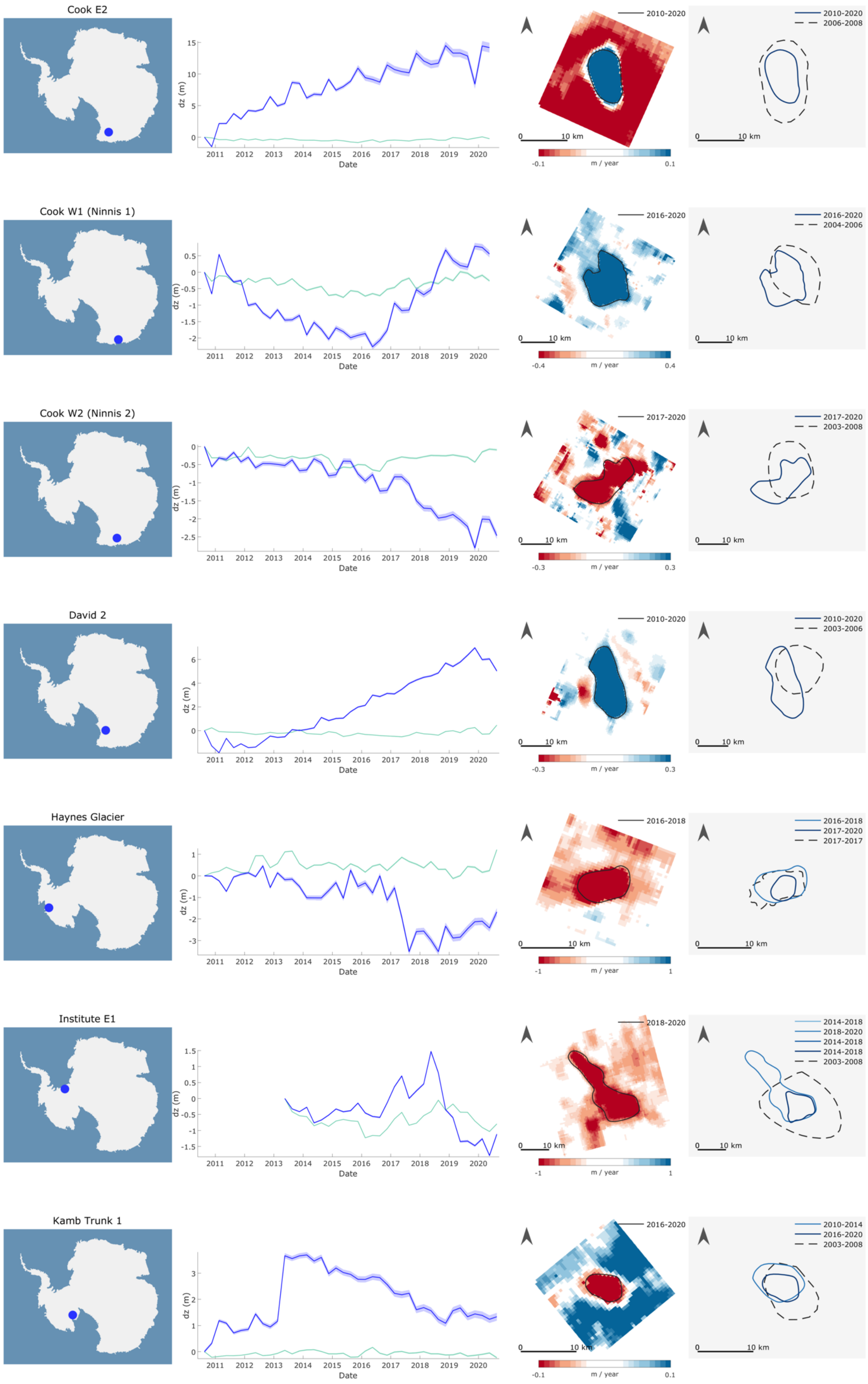

Cook E2
dz (m)
Date
2010-2020
2006-2008
10 km
m / year
Cook W1 (Ninnis 1)
2016-2020
2004-2006
Cook W2 (Ninnis 2)
2017-2020
2003-2008
David 2
2010-2020
2003-2006
Haynes Glacier
2016-2018
2017-2020
2017-2017
Institute E1
2018-2020
2014-2018
2018-2020
2014-2018
2014-2018
2003-2008
Kamb Trunk 1
2016-2020
2010-2014
2016-2020
2003-2008
2011
2012
2013
2014
2015
2016
2017
2018
2019
2020

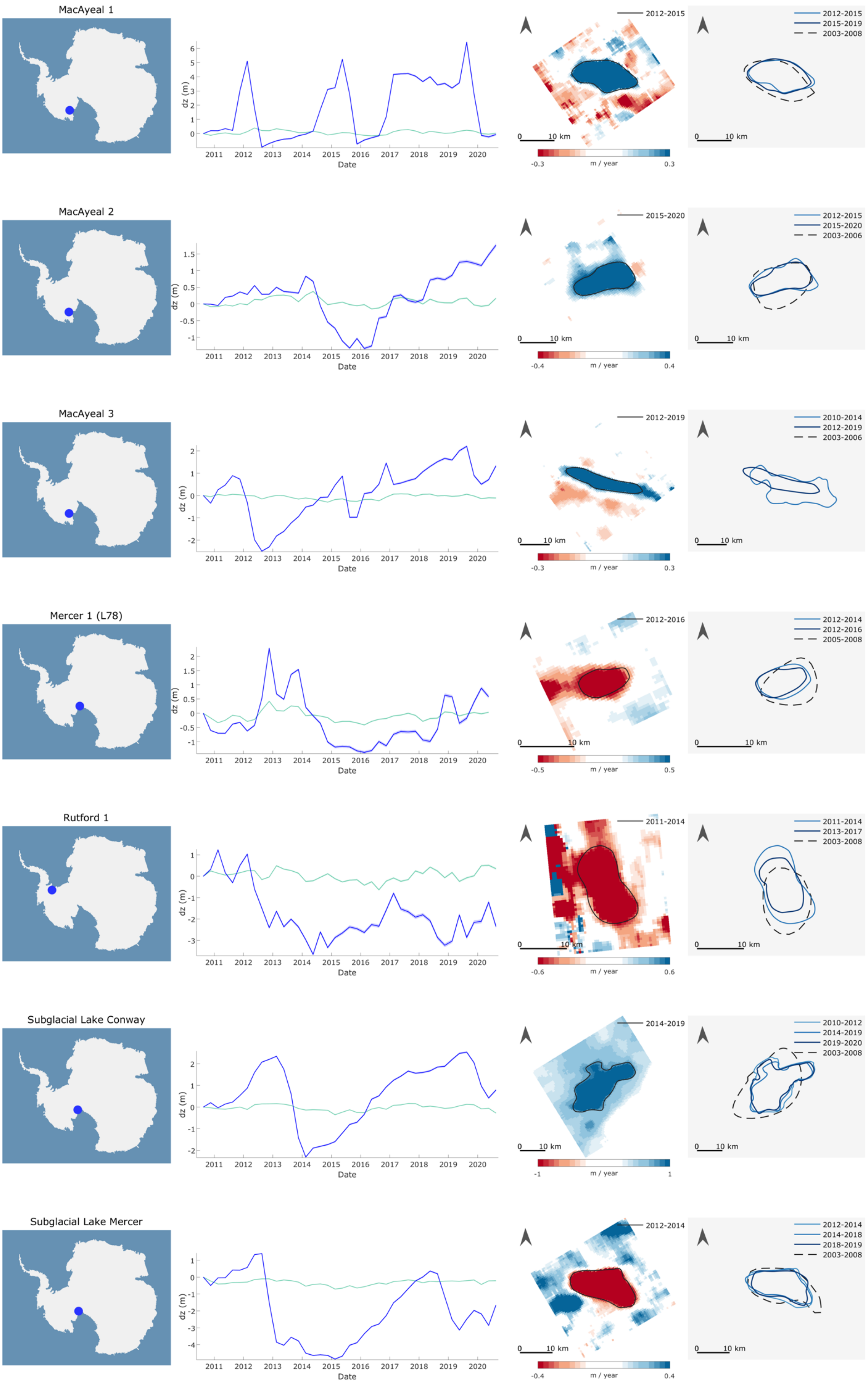
MacAyeal 1
dz (m)
Date
2011 2012 2013 2014 2015 2016 2017 2018 2019 2020
2012-2015
2012-2015
2015-2019
2003-2008
0 10 km
-0.3 m / year 0.3
MacAyeal 2
2015-2020
2012-2015
2015-2020
2003-2006
-0.4 m / year 0.4
MacAyeal 3
2012-2019
2010-2015
2012-2019
2003-2006
-0.3 m / year 0.3
Mercer 1 (L78)
2012-2016
2012-2014
2012-2016
2005-2008
-0.5 m / year 0.5
Rutford 1
2011-2014
2011-2014
2013-2017
2003-2008
-0.6 m / year 0.6
Subglacial Lake Conway
2014-2019
2010-2012
2014-2019
2019-2020
2003-2008
-1 m / year 1
Subglacial Lake Mercer
2012-2014
2012-2014
2014-2018
2018-2019
2003-2008
-0.4 m / year 0.4

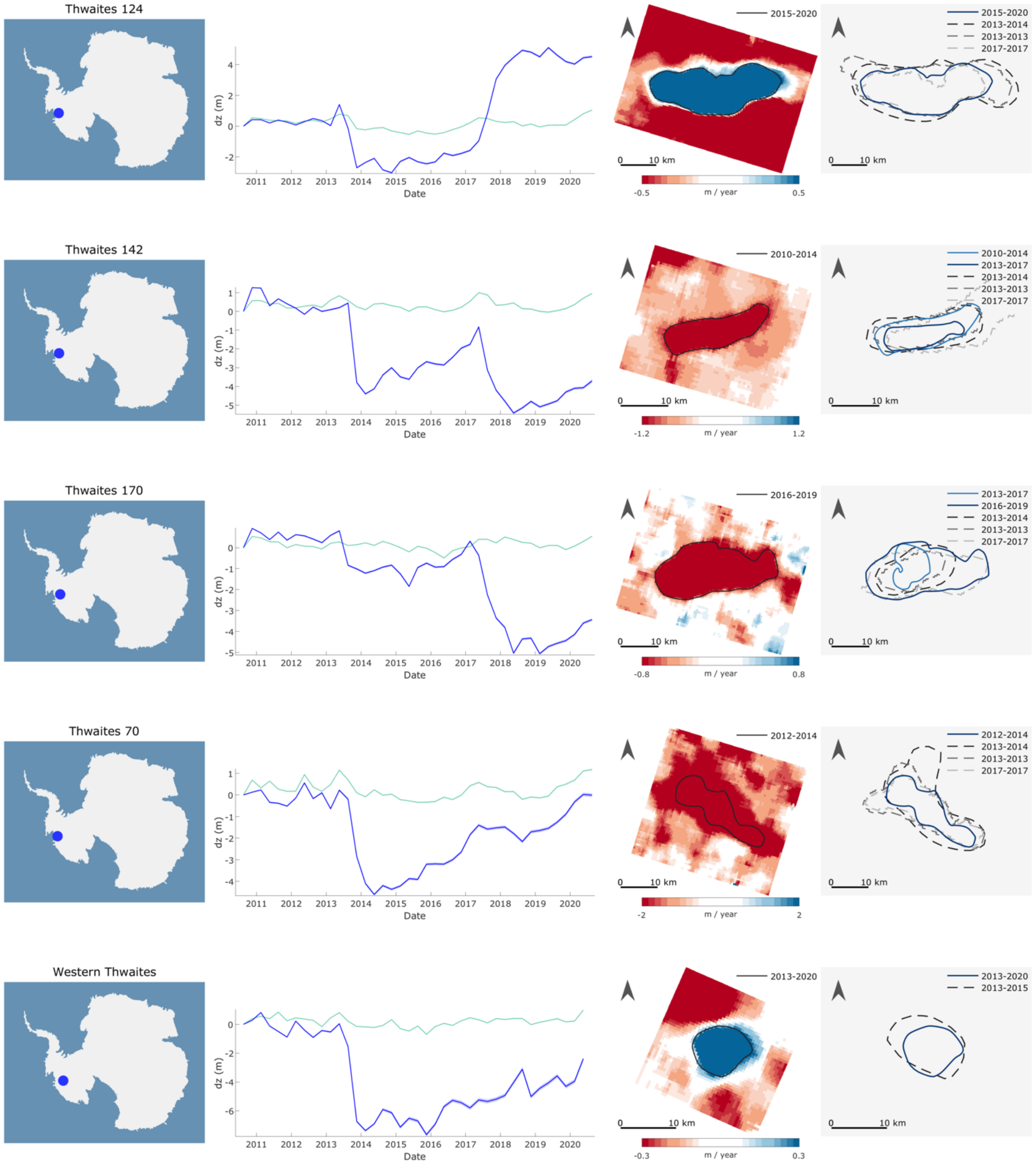

Supplementary Figure 3. **Location map, elevation change time series, an elevation change map and time-variable outlines for 19 subglacial lakes in the existing archive (from left to right).** For each subglacial lake (row) we show: (a) a map showing the subglacial lake location (blue dot) on the Antarctic Ice Sheet. (b) Three-month mean timeseries of ice surface elevation change (dz) from 2010 to 2020, with subglacial lake elevation change (blue line) compared to elevation change from the non-subglacial lake region (green line). Cumulative error is shown by the shaded area around each time series. (c) A surface elevation change map is shown for one event on each subglacial lake, with the corresponding lake boundary delineated from these data (black line). Time periods for these maps and boundaries are illustrated in the top right corner, and the North direction is indicated by the grey arrow. (d) Time-variable subglacial lake boundaries (light to dark blue solid lines) are delineated for each active phase in this study, with existing lake boundaries also shown (light to dark grey dashed lines) where they exist (Smith et al., 2009; Smith et al., 2017; Malczyk et al., 2020; Hoffman et al., 2020).

Subglacial Lake	Latitude (°)	Longitude (°)	Ice Sheet	GL distance (km)	MEaSUREs ice speed (m/yr)	BedMachine ice thickness (m)
Academy_45	-84.22	-59.07	EAIS	45	183	2586
Academy_91	-84.34	-55.70	EAIS	91	80	2001
Adams_17	-67.02	110.09	EAIS	17	151	1354
Adams_19	-67.09	109.65	EAIS	19	113	1703
Amery_22	-72.70	66.28	EAIS	22	76	982
ANZAC_19	-67.08	109.42	EAIS	19	58	1534
Beardmore_107	-84.70	168.32	EAIS	107	172	1346
Beardmore_18	-83.91	171.67	EAIS	18	142	807
Beardmore_66	-84.45	171.33	EAIS	66	117	1496
Beaver_27	-67.13	51.64	EAIS	27	291	1238
Borchgrevink_188	-72.60	18.57	EAIS	188	29	1145
Borchgrevink_198	-72.57	21.48	EAIS	198	49	596
Byrd_10	-80.51	157.64	EAIS	10	681	2086
Byrd_97	-80.62	153.13	EAIS	97	189	1395
Byrd_98	-81.13	154.43	EAIS	98	109	1528
CookEast_134	-70.17	153.91	EAIS	134	39	1993
CookEast_156	-70.48	154.40	EAIS	156	12	2098
CookWest_186	-70.20	149.29	EAIS	186	44	2661
CookWest_58	-69.19	151.08	EAIS	58	125	1912
CookWest_67	-69.21	150.20	EAIS	67	93	2561
David_180	-74.43	155.52	EAIS	180	10	1964
David_80	-75.17	157.93	EAIS	80	110	2066
Evans_158	-74.80	-70.58	WAIS	158	32	795
Fisher_168	-73.24	61.43	EAIS	168	79	345
Français_36	-66.96	138.15	EAIS	36	86	1132
Français_46	-67.04	138.11	EAIS	46	66	1223
Frost_28	-67.29	129.67	EAIS	28	222	978
Institute_134	-82.02	-78.64	WAIS	134	51	1654
Institute_14	-81.08	-75.52	WAIS	14	190	1503
Institute_142	-82.19	-75.27	WAIS	142	97	1768

Institute_171	-82.44	-76.88	WAIS	171	83	1604
Institute_176	-82.48	-76.86	WAIS	176	84	1821
Jutulstraumen_69	-72.51	-1.74	EAIS	69	215	1795
Jutulstraumen_71	-72.54	-1.47	EAIS	71	220	2204
Jutulstraumen_74	-72.67	-0.90	EAIS	74	226	1738
KingBaudouinEast_18	-71.21	26.48	EAIS	18	95	860
KingBaudouinEast_3	-70.64	28.94	EAIS	3	45	623
KingBaudouinWest_51	-70.98	29.90	EAIS	51	18	849
Kronshtadskiy_95	-71.58	74.18	EAIS	95	54	1472
Kyoto_96	-75.65	-125.37	WAIS	96	65	1488
Lambert_118	-74.40	67.17	EAIS	118	76	1793
Lambert_119	-74.33	69.38	EAIS	119	178	3220
Lambert_152	-74.45	70.07	EAIS	152	148	2552
Lambert_45	-73.77	67.67	EAIS	45	322	2745
Lambert_84	-74.07	68.79	EAIS	84	277	2970
Lambert_99	-74.22	67.25	EAIS	99	103	1689
Lazarev_35	-70.68	14.00	EAIS	35	15	1010
MacAyeal_299	-77.57	-131.59	WAIS	299	20	2169
Matusevich_53	-69.86	157.48	EAIS	53	178	1316
Matusevich_57	-69.89	157.45	EAIS	57	177	1429
Mellor_112	-74.33	66.16	EAIS	112	195	2595
Mellor_183	-74.61	63.33	EAIS	183	52	2680
Mill_161	-85.58	171.03	EAIS	161	47	1184
Mill_165	-85.61	170.47	EAIS	165	40	1366
MoscowUniversity_19	-67.56	119.01	EAIS	19	301	1030
MoscowUniversity_194	-69.08	117.01	EAIS	194	41	2547
MoscowUniversity_37	-67.74	119.02	EAIS	37	211	1381
MoscowUniversity_44	-67.67	118.07	EAIS	44	287	1971
MoscowUniversity_60	-67.91	119.47	EAIS	60	129	1503
MoscowUniversity_62	-67.79	117.99	EAIS	62	246	1739
Mulock_60	-78.69	158.49	EAIS	60	87	1165
Ninnis_160	-69.86	146.19	EAIS	160	58	2670

NinnisEast_10	-68.58	148.20	EAIS	10	229	945
NinnisEast_63	-69.06	148.32	EAIS	63	47	1885
Recovery_50	-81.09	-33.34	EAIS	50	73	1829
Recovery_68	-81.03	-30.50	EAIS	68	102	1904
Rutford_192	-76.99	-89.14	WAIS	192	38	2386
Scott_12	-85.56	-152.72	EAIS	12	366	1374
Scott_19	-85.64	-152.92	EAIS	19	266	1455
Stockholm_72	-75.36	-120.03	WAIS	72	133	1615
Stockholm_77	-75.39	-120.02	WAIS	77	137	1498
SupportForce_153	-84.02	-47.65	EAIS	153	111	1775
SupportForce_3	-82.60	-46.41	EAIS	3	266	1288
Totten_191	-69.15	112.49	EAIS	191	55	2585
Totten_35	-67.82	113.79	EAIS	35	427	1466
Totten_36	-67.60	112.89	EAIS	36	312	1712
Totten_40	-67.77	113.30	EAIS	40	340	1441
Totten_52	-67.58	112.62	EAIS	52	181	1644
Totten_7	-67.58	113.89	EAIS	7	528	1456
Totten_82	-67.91	112.21	EAIS	82	172	1841
Vanderford_12	-66.82	110.70	EAIS	12	513	1298
Vanderford_4	-66.72	110.77	EAIS	4	206	1898
Vanderford_5	-66.76	110.73	EAIS	5	439	1504
Vigrid_4	-70.56	7.92	EAIS	4	77	755
Whillans_180	-83.53	-140.06	WAIS	180	394	1072

Subglacial Lake	Start date	End date	Elev. change (m)	Area (km^2)	Volume change (km^3)	Start date	End date	Elev. change (m)	Area (km^2)	Volume change (km^3)	Start date	End date	Elev. change (m)	Area (km^2)	Volume change (km^3)	Start date	End date	Elev. change (m)	Area (km^2)	Volume change (km^3)	Start date	End date	Elev. change (m)	Area (km^2)	Volume change (km^3)
Academy_45	2013.9	2016.1	-2	395.82	-0.79	2016.1	2018.5	3.5			2018.5	2020.7	-1												
Academy_91	2013.4	2013.9	5.5			2013.9	2015.1	-4.5			2015.1	2020.1	8	238.07	1.90	2020.1	2020.7	-2							
Adams_17	2010.7	2014.1	5	57.47	0.29	2014.1	2019	-6	74.53	-0.45															
Adams_19	2014	2020	-4.5																						
Amery_22	2012.7	2017	-4.5																						
ANZAC_19	2014.2	2020.7	-5																						
Beardmore_107	2010.7	2011.7	-2			2011.7	2020.7	5.5	41.76	0.23															
Beardmore_18	2010.7	2020.1	3.5	13.62	0.05																				
Beardmore_66	2010.9	2020.1	1.5	18.09	0.03																				
Beaver_27	2018	2019.9	2.5			2019.8	2020.7	-1.5																	
Borchgrevink_188	2010.7	2017	-2.5																						
Borchgrevink_198	2010.7	2015.3	-6	78.49	-0.47	2016	2020.7	3.5	55.50	0.19															
Byrd_10	2010.7	2011.7	-2			2011.7	2016.1	5	196.09	0.98	2016.1	2020.7	-3.5	112.95	-0.40										
Byrd_97	2010.7	2011	-1			2014.1	2018	3			2020.3	2020.7	1.5												
Byrd_98	2011.1	2020	5	24.44	0.12																				
CookEast_134	2012.7	2020.7	4	27.34	0.11																				
CookEast_156	2014.3	2019	4																						
CookWest_186	2010.9	2011.9	-2			2015	2020.7	3.5	55.40	0.19															
CookWest_58	2017	2020.7	-3.5																						
CookWest_67	2010.7	2011.4	2.5			2012.1	2016.1	-6	219.05	-1.31	2016.1	2020.7	4.5	218.36	0.98										
David_180	2010.7	2011.9	-1.2			2011.9	2014.9	1.5	28.26	0.04	2014.9	2016.7	-4			2016.7	2020.7	3.5	33.01	0.12					
David_80	2010.9	2012.3	2.5			2012.3	2020.7	-6.5	95.99	-0.62															
Evans_158	2010.7	2020.7	4	8.49	0.03																				
Fisher_168	2010.7	2014.2	2	33.93	0.07	2014.2	2015.5	-4	128.97	-0.52															
Français_36	2010.7	2019.7	5	15.84	0.08	2019.7	2020.7	-1																	
Français_46	2011.2	2020	-5	9.72	-0.05																				
Frost_28	2010.7	2013.3	-5	53.64	-0.27	2013.3	2020.7	6	57.10	0.34															

Institute_134	2014	2018.9	3																	
Institute_14	2013.3	2018.3	2.7	49.97	0.13	2018.3	2020.7	-1.5	49.78	-0.07										
Institute_142	2016.1	2018.1	2.3	27.09	0.06	2018.1	2020.5	-2.3	36.94	-0.08										
Institute_171	2017.6	2020	-3.5	26.05	-0.09															
Institute_176	2018	2019.9	1.8	19.99	0.04	2019.9	2020.7	-0.7												
Jutulstraumen_69	2017	2020.7	-3	30.82	-0.09															
Jutulstraumen_71	2013.9	2015.3	-4			2015.3	2020.7	3	50.03	0.15										
Jutulstraumen_74	2014	2015.5	-5			2015.5	2020.7	3	223.84	0.67										
KingBaudouinEast_18	2010.7	2012.2	1			2012.2	2016	-2.5												
KingBaudouinEast_3	2013.5	2016.1	-7			2016.1	2018.7	3			2018.7	2020.7	-5							
KingBaudouinWest_51	2015	2020.7	4	19.74	0.08															
Kronshtadskiy_95	2011.7	2015	3	35.20	0.11	2015	2020	-3	33.50	-0.10										
Kyoto_96	2017	2020.1	-3	24.43	-0.07	2020.1	2020.7	2												
Lambert_118	2014.1	2020.7	4	26.38	0.11															
Lambert_119	2010.7	2011.5	-0.8			2011.5	2015	5	202.86	1.01	2015	2017.1	-4			2018	2020.3	2.5	146.97	0.37
Lambert_152	2012	2015.9	-2.5	125.58	-0.31	2015.9	2020.7	2.5	67.19	0.17										
Lambert_45	2015.3	2018.8	3	179.59	0.54															
Lambert_84	2010.7	2014.1	-3	121.42	-0.36	2015	2017.5	8			2017.5	2020.7	-3	110.55	-0.33					
Lambert_99	2010.7	2020.7	4.5	31.10	0.14															
Lazarev_35	2014	2020	2.5																	
MacAyeal_299	2012.3	2020.7	-3	18.46	-0.06															
Matusevich_53	2012	2020.7	-7	12.49	-0.09															
Matusevich_57	2011.7	2018.1	6	12.29	0.07	2018.1	2020.4	-2												
Mellor_112	2011.9	2013	-2.5	92.36	-0.23	2013	2014.5	1.5			2018.8	2020.7	-3	58.77	-0.18					
Mellor_183	2013	2016.3	4			2016.3	2017.1	-3			2017.1	2020.2	3.5			2020.2	2020.7	-4		
Mill_161	2015	2018.9	2	5.37	0.01	2018.9	2020.3	-1.3	5.18	-0.01										
Mill_165	2010.7	2013.1	2.7	26.56	0.07	2013.1	2015.6	-3.6	35.79	-0.13	2016.7	2020.7	3	17.19	0.05					
MoscowUniversity_19	2015	2020.1	-10																	
MoscowUniversity_194	2012.7	2017.4	-3.5			2018	2020.7	1												
MoscowUniversity_37	2010.7	2012.7	-3			2016.1	2018	-6			2018	2020.7	3	36.63	0.11					
MoscowUniversity_44	2010.7	2012	-5			2012	2016.9	9			2016.9	2017.4	-4			2017.4	2020.7	6		
MoscowUniversity_60	2016	2019	3																	

MoscowUniversity_62	2013	2019.3	6.5			2019.3	2020.1	-3																	
Mulock_60	2011.1	2019	4.5	14.03	0.06																				
Ninnis_160	2011.1	2018.9	-4	75.69	-0.30																				
NinnisEast_10	2013	2018	-8																						
NinnisEast_63	2010.7	2019	-3	60.39	-0.18	2019	2020.3	-5			2020.3	2020.7	1												
Recovery_50	2012	2013.3	-2			2013.3	2017.9	1			2017.9	2020.1	-5.5												
Recovery_68	2012.3	2019	3.5	15.97	0.06																				
Rutford_192	2017.1	2018.1	-4			2018.1	2020	2	25.83	0.05															
Scott_12	2010.9	2013.4	1			2013.3	2014.1	-3			2014.1	2016.7	3	59.44	0.18	2016.7	2018.1	-3.2	32.96	-0.11	2018	2020	4.2	29.77	0.13
Scott_19	2010.7	2013.1	4.5	49.33	0.22	2013.1	2020.1	-5.5	14.70	-0.08	2020.1	2020.7	2.5												
Stockholm_72	2014.7	2017.4	4			2017.4	2019	-2	40.75	-0.08															
Stockholm_77	2013.3	2017.9	-4	23.00	-0.09																				
SupportForce_153	2016.1	2017.3	-2.3	19.28	-0.04	2017.3	2020.7	1.5	11.72	0.02															
SupportForce_3	2010.7	2015.1	-3			2015.1	2020.7	4.5																	
Totten_191	2011	2017	-2																						
Totten_35	2016.1	2019.4	7			2019.4	2020	-2.5																	
Totten_36	2011.9	2012.6	-5			2012.6	2020.2	8	62.21	0.50															
Totten_40	2016	2017.6	-6			2017.6	2020.7	3																	
Totten_52	2010.9	2013.4	-8.4	45.63	-0.38	2013.4	2017.4	8			2017.4	2018	-1			2018	2020.7	3							
Totten_7	2010.7	2012.3	3			2012.3	2012.9	-9			2012.9	2016.8	8												
Totten_82	2014	2020.7	-4.5																						
Vanderford_12	2011	2020.7	-9.5	7.66	-0.07																				
Vanderford_4	2011.2	2017	7	17.27	0.12	2017	2020	-3																	
Vanderford_5	2011	2020.7	-7	23.66	-0.17																				
Vigrid_4	2014	2018	3																						
Whillans_180	2011.7	2013.7	2.5	179.90	0.45	2013.7	2015.1	-1.8	118.50	-0.21	2015.4	2016.4	2	102.99	0.21	2016.4	2019.9	-1			2020	2021	1.7	150.52	0.26

Supplementary Table 1. All (85) new subglacial lakes detected in this study, metadata and activity log.

Wilson et al., 2025

Subglacial Lake	Latitude (°)	Longitude (°)	Ice Sheet	MEaSUREs ice speed (m/yr)	BedMachine ice thickness (m)	Activity ->	Start date	End date	Elev. change (m)	Area (km^2)	Volume change (km^3)
Subglacial Lake Mercer (Mercer 2)	-84.66	-149.68	WAIS	257	1188		2011	2012.8	1.5		
							2012.8	2015.1	-6.5	154.54	-1.00
							2015.1	2018.3	6	127.99	0.77
							2018.3	2019.3	-3.7		
Subglacial Lake Conway (Whillans 4)	-84.37	-148.72	WAIS	377	909		2011	2013.1	2.5	323.57	0.81
							2013.1	2014.1	-4.7		
							2014.1	2019.7	5	253.88	1.27
							2019.7	2020.5	-2.5		
MacAyeal 1	-79.95	-145.36	WAIS	424	1096		2011.8	2012.2	4.5		
							2012.2	2012.6	-5.5		
							2012.6	2015.4	6	115.72	0.69
							2015.4	2015.9	6		
							2015.9	2017.1	5		
							2017.1	2019.1	-1		
							2019.1	2019.7	3		
							2019.7	2020.2	-6		
MacAyeal 2	-79.83	-144.08	WAIS	399	1254		2011.2	2014.2	1		
							2014.2	2015.7	-2.5	112.28	-0.28
							2016.1	2020.5	3.5	94.08	0.33
MacAyeal 3	-80.02	-142.81	WAIS	377	1229		2010.7	2011.7	1		
							2011.7	2012.7	-3.5		
							2012.7	2015.3	3.5		
							2015.3	2015.7	-2		
							2015.7	2019.7	3		
							2019.7	2020.2	-1.5		
Thwaites 170 (THW170)	-76.87	-106.00	WAIS	151	2555		2013.2	2013.9	-4.5		
							2013.9	2017.2	3.5	80.88	0.28
							2017.2	2018.3	-8.5	327.86	-2.79
							2018.3	2020.5	3.5		
Thwaites 142 (THW142)	-76.63	-106.18	WAIS	227	2288		2013.5	2014	-5.3	119.15	-0.63
							2014	2017.4	3	62.33	0.19
							2017.4	2018.3	-4.5		
							2018.3	2020.5	1.5		
Thwaites (THW124)	-76.48	-106.95	WAIS	299	2204		2013.4	2014.9	-4		
							2014.9	2018.7	8		
Thwaites 70 (THW70)	-76.01	-107.08	WAIS	360	2199		2013	2014.4	-4	161.23	-0.64

							2014.4	2020.5	4		
Haynes Glacier (HG)	-75.90	-110.68	WAIS	98	1855		2017	2017.7	-7	47.94	-0.34
							2017.7	2020.1	3.7	16.73	0.06
Thwaites West (TW)	-77.19	-113.81	WAIS	75	2729		2013	2014.1	-7		
							2014.1	2020.5	3.5		
Cook E2	-72.80	155.79	EAIS	3	2778		2010.7	2020.5	15	63.49	0.95
Cook W1 (Ninnis 1)	-69.66	149.71	EAIS	53	2716		2010.7	2011	1		
							2011	2016.3	-3		
							2016.7	2020.5	3.5	142.49	0.50
Cook W2 (Ninnis 2)	-70.84	149.38	EAIS	25	2842		2015.7	2019.9	-2.5		
Institute E1	-82.13	-74.49	WAIS	99	2056		2014.3	2018.3	2.5	73.55	0.18
									2.5	27.15	0.07
							2018.3	2020.5	-4	209.39	-0.84
KT1 (Kamb Trunk 1)	-81.95	-140.40		4	1106		2010.7	2014.8	3.7	44.47	0.16
							2016.7	2020.5	2.4	24.05	0.06
L78 (Mercer 1)	-84.60	-154.19	WAIS	329	895		2012.1	2012.9	2.5	42.74	0.11
							2012.9	2016	-3.2	34.99	-0.11
							2016.4	2019	1.7		
Rutford 1	-78.18	-84.17	WAIS	382	2443		2011	2014.4	-6	129.81	-0.78
							2014.9	2017.1	4	76.64	0.31
							2017.1	2018.9	-3.5		
							2018.9	2020.5	2		
David 2	-75.33	155.52	EAIS	47	2097		2010.7	2011.1	-2		
							2011.1	2019.8	8.5	181.00	1.54
							2019.8	2020.5	-2		

Supplementary Table 2. Analysis of previously detected subglacial lakes (Smith et al., 2009; Smith et al., 2017), metadata and activity log.
Wilson et al., 2025
